# Tiliroside Suppresses Uric Acid Production in Hepatocytes and Attenuates Purine-Induced Hyperuricemia in Male ICR Mice

**DOI:** 10.3390/molecules30193914

**Published:** 2025-09-28

**Authors:** Shin-ichi Adachi, Shinji Kondo, Yusuke Sato, Fumiaki Yoshizawa, Kazumi Yagasaki

**Affiliations:** 1 Faculty of Health Sciences for Welfare, Kansai University of Welfare Sciences, Kashiwara 582-0026, Osaka, Japan; 2 Alliance for Research on the Mediterranean and North Africa (ARENA), University of Tsukuba, Tsukuba 305-8572, Ibaraki, Japan; kondo.shinji.ga@u.tsukuba.ac.jp; 3 School of Agriculture, Tokai University, Mashiki 861-2205, Kumamoto, Japan; sato.yusuke.k@tokai.ac.jp; 4 School of Agriculture, Utsunomiya University, Utsunomiya 321-8505, Tochigi, Japan; fumiaki@cc.utsunomiya-u.ac.jp; 5 United Graduate School of Agricultural Science, Tokyo University of Agriculture and Technology, Fuchu 183-8509, Tokyo, Japan; yagasaki@cc.tuat.ac.jp

**Keywords:** tiliroside, AML12 hepatocytes, uric acid, hyperuricemia, gout, purine body, xanthine oxidase

## Abstract

Tiliroside (kaempferol-3-O-(6′′-p-coumaroyl)-glucoside), a flavonoid glycoside found in rose hips and berries, has been reported to possess various bioactivities. This study aimed to evaluate its antihyperuricemic potential by assessing direct xanthine oxidase (XO) inhibitory activity, suppression of uric acid (UA) production in AML12 hepatocytes, and efficacy in male ICR mice with purine nucleotide-induced hyperuricemia. XO inhibition was evaluated using a UV absorbance-based assay, and UA production was measured in hepatocytes stimulated with UA precursors. Mice were orally administered tiliroside for three days prior to purine nucleotide injection. Although tiliroside exhibited weak XO inhibition (IC_50_ > 100 µM), it significantly suppressed UA production in hepatocytes in a concentration-dependent manner. In hyperuricemic mice, tiliroside (300 mg/kg) lowered plasma and hepatic UA levels by approximately 30% and 55%, respectively (*p* < 0.05). Hepatic XO activity was significantly decreased, while XO protein expression remained unchanged. Furthermore, mRNA levels of urate transporter 1 (URAT1) were significantly decreased in the kidney of tiliroside-treated hyperuricemic mice. These findings suggest that tiliroside exerts antihyperuricemic effects by suppressing UA production in the liver and modulating renal UA reabsorption. Tiliroside may serve as a beneficial dietary compound with bioactivity for the prevention and management of hyperuricemia and gout.

## 1. Introduction

Hyperuricemia is a condition marked by excessive accumulation of uric acid (UA) in the bloodstream, primarily resulting from excessive UA synthesis in the liver and/or insufficient excretion via the kidneys [1]. It is a known risk factor for gout and kidney disease and is associated with metabolic syndrome, hypertension, and cardiovascular disease [2,3]. A high dietary intake of purine-rich foods such as meats and seafood is associated with an increased risk of gout [4]. UA is produced mainly in the liver via purine metabolism catalyzed by xanthine oxidase (XO), and excreted by the kidneys through transporters such as URAT1, GLUT9, and ABCG2. Currently available UA-lowering drugs, including allopurinol, febuxostat, and benzbromarone, function by inhibiting XO activity or modulating UA transporters, but they may cause side effects such as allergic reactions, diarrhea, and hepatotoxicity [5,6,7]. Given the limitations of current therapies, novel and safe compounds from foods and natural resources are expected to be discovered to suppress hyperuricemia.

Tiliroside (kaempferol-3-O-(6′′-p-coumaroyl)-glucoside), a naturally occurring flavonoid glycoside with a polyphenolic structure, is abundant in fruits such as rose hips, strawberries, and raspberries [8,9,10]. Structurally, tiliroside is a derivative of kaempferol, a dietary flavonol widely found in apples, beans, carrots, and saffron. Kaempferol has been reported to exert antihyperuricemic, antioxidative, and anti-inflammatory effects [11,12]. Previous studies have highlighted the diverse bioactivities of tiliroside, including antioxidative, anti-inflammatory, hepatoprotective, glucose-lowering, antiobesity, and anti-aging properties [13,14]. These biological activities suggest that tiliroside may serve as a promising therapeutic agent for the prevention and management of metabolic disorders, particularly hyperuricemia. Several polyphenols, such as urolithin A, baicalein, and fisetin, are reportedly effective against hyperuricemic model mice [15,16,17]. Moreover, a previous study has shown that quercetin, another polyphenol, can reduce plasma UA levels in pre-hyperuricemic individuals [18]. However, whether tiliroside possesses hypouricemic properties remains unclear at present.

To date, the search for novel food-derived compounds with antihyperuricemic activity has frequently employed in vitro assays that directly measure XO inhibitory activity. The results obtained from these assays have made a significant contribution to assessing the in vivo efficacy of candidate compounds and remain a valuable tool for initial screening [19,20]. However, such assays may overlook novel dietary and natural substances that act on targets other than XO, as well as intracellular metabolites derived from test compounds. To address this limitation, we employed a hepatocyte-based assay using AML12 cells, which enables the evaluation of UA production in a physiologically relevant context [21]. Combined with a purine-induced hyperuricemic mouse model, this approach allows for a more comprehensive assessment of the UA-lowering potential of candidate compounds.

**Figure 1:**
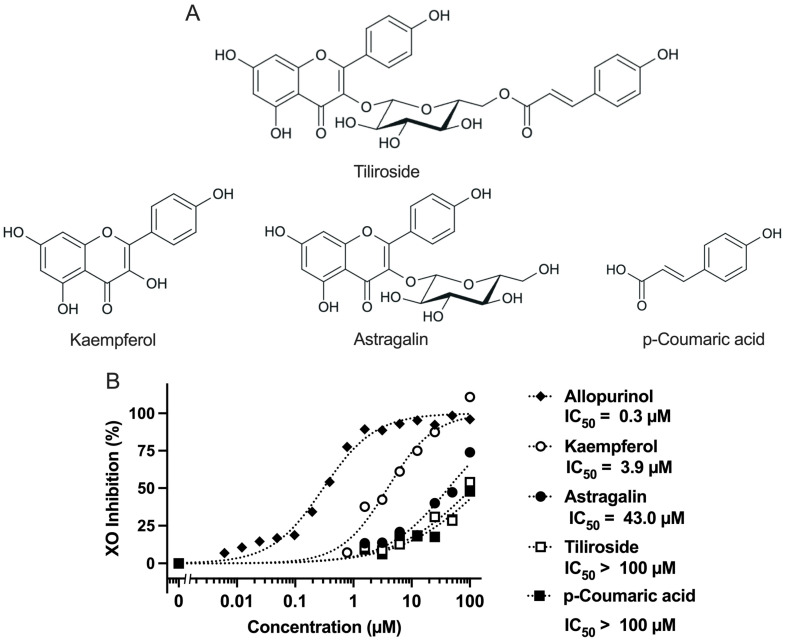
Chemical structures of tiliroside, kaempferol, astragalin, and p-coumaric acid (**A**) and their direct inhibitory effects on XO activity (**B**). Direct XO inhibitory activity in vitro was evaluated based on mean values from three independent experiments. IC_50_ values were estimated by nonlinear regression using dose–response curves generated from at least seven concentrations of each compound. Allopurinol was included as a positive control and is shown in panel (**B**).

Therefore, this study aimed to comprehensively evaluate the antihyperuricemic effect of tiliroside by investigating its ability to suppress UA production in AML12 hepatocytes and reduce UA levels in a mouse model of purine-induced hyperuricemia. To elucidate the underlying mechanisms, hepatic UA concentrations, XO activity, and renal mRNA expression of urate transporter-related genes (URAT1, GLUT9, and ABCG2) were analyzed. In addition, the direct XO inhibitory activity of tiliroside was assessed in vitro to determine whether its UA-lowering effect is attributable to XO inhibition. As a secondary objective, we examined the structure–activity relationship of tiliroside by comparing its effects on UA production in hepatocytes and direct XO inhibition with those of three structurally related polyphenols: kaempferol (its aglycone), astragalin (kaempferol-3-O-glucoside), and p-coumaric acid (a hydroxycinnamic acid moiety esterified to the glucose unit of tiliroside). Astragalin is a flavonoid glycoside found in edible fruits such as mulberries and raspberries and has been reported to exhibit anti-inflammatory and antioxidant activities [22]. These compounds were selected to clarify the contribution of each structural component to the overall bioactivity of tiliroside, particularly in terms of UA production and XO inhibition. The chemical structures of tiliroside and its related polyphenols are shown in Figure 1A.

## 2. Results

### 2.1. Direct Inhibitory Effects of Test Compounds on XO Activity In Vitro

Tiliroside, kaempferol, astragalin, p-coumaric acid, and allopurinol were evaluated for their XO inhibitory activity (Figure 1B). Allopurinol, a well-known potent XO inhibitor used as a positive control, showed an IC_50_ value of 0.3 µM, indicating the reliability of the assay system employed. Kaempferol exhibited potent inhibitory activity with an IC_50_ of 3.9 µM. The IC_50_ values were 43 µM for astragalin and >100 µM for both tiliroside and p-coumaric acid.

### 2.2. Viability and UA Production in AML12 Hepatocytes Following Treatment with Test Compounds

**Figure 2:**
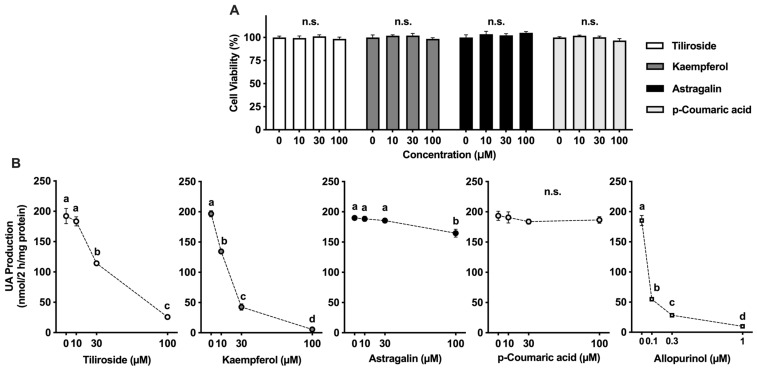
Effects of tiliroside, kaempferol, astragalin, and p-coumaric acid on cell viability (**A**) and UA production (**B**) in AML12 hepatocytes. Panel (**A**): Cell viability was normalized to the DMSO-treated control group and expressed as mean ± SEM for six replicate wells, shown as a percentage. Panel (**B**): UA production data are presented as mean ± SEM from six replicate wells. Allopurinol was included as a positive control and is shown in panel (**B**). Values not sharing a common letter among the same compounds are significantly different at *p* < 0.05 (Tukey’s test).

The effects of tiliroside, kaempferol, astragalin, and p-coumaric acid on the viability of AML12 hepatocytes were evaluated using the WST-8 assay. Treatment with these compounds did not exhibit any cytotoxic effects at concentrations up to 100 µM (Figure 2A). Accordingly, this concentration was applied in subsequent experiments to examine the compounds’ effects on UA production under conditions that maintain cell viability.

Figure 2B shows the effects of allopurinol, tiliroside, kaempferol, astragalin, and p-coumaric acid on UA production in AML12 cells. Allopurinol and kaempferol significantly and dose-dependently reduced UA production at concentrations of 0.1, 0.3, and 1 µM and 10, 30, and 100 µM, respectively, compared to control (0 µM), with maximum reductions of approximately 94.6% at 1 µM for allopurinol and 97.2% at 100 µM for kaempferol. Similarly, tiliroside significantly and dose-dependently reduced UA production at 30 and 100 µM, with a maximum reduction of approximately 86.6% at 100 µM. Astragalin significantly reduced UA production at a concentration of 100 µM, with a maximum reduction of approximately 13.3%. UA production remained unaffected by p-coumaric acid at concentrations up to 100 µM.

### 2.3. Effect of Tiliroside on Antihyperuricemic Activity in Mice

**Figure 3:**
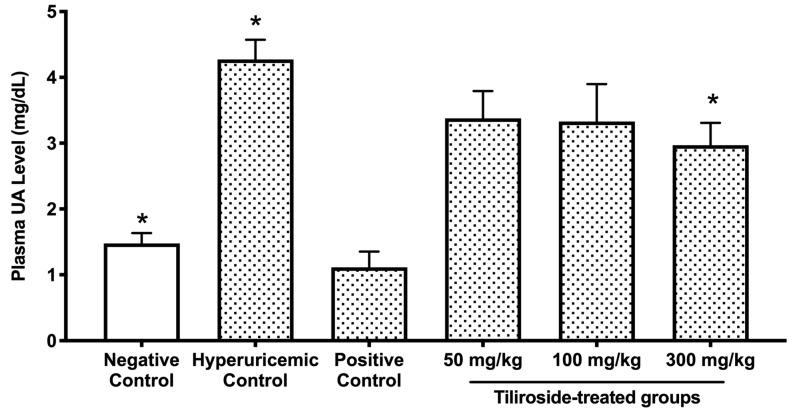
Tiliroside administration reduces plasma UA levels in mice with purine-induced hyperuricemia. Mice received oral doses of tiliroside at the indicated concentrations for three consecutive days. Hyperuricemia was induced by intraperitoneal injection of IMP and GMP (300 mg/kg each) one hour after the final oral administration. Negative and hyperuricemic control groups received 0.5% CMC-Na instead of test compounds; the negative group was injected with PBS (–) instead of purine nucleotides. Allopurinol (10 mg/kg body weight) was used as the positive control. Data are expressed as mean ± SEM for 8–10 mice, with duplicate measurements per mouse. * *p* < 0.05 versus the hyperuricemic control group (Dunnett’s test).

Intraperitoneal co-administration of GMP and IMP led to a significant elevation in plasma UA levels in mice, showing a statistically significant difference between the hyperuricemic control group and the negative control group that received PBS (–) alone (Figure 3). Oral treatment with allopurinol (10 mg/kg body weight, positive control) or tiliroside (300 mg/kg body weight) significantly reduced this UA increase. In contrast, lower doses of tiliroside (50 and 100 mg/kg body weight) failed to produce a statistically significant reduction in plasma UA levels.

### 2.4. Hepatic UA Concentration and XO Activity and Protein Expression Following Tiliroside Treatment in Hyperuricemic Mice

The hepatic UA concentration was significantly elevated in the hyperuricemic control group compared to the negative control group. Oral administration of allopurinol (10 mg/kg, positive control) and tiliroside at doses of 100 and 300 mg/kg effectively reduced hepatic UA levels relative to the hyperuricemic control group (Table 1). In contrast, the 50 mg/kg dose of tiliroside did not produce a significant reduction.

The hepatic XO activity in the hyperuricemic control group was significantly higher than that in negative control group. The XO activities in allopurinol (10 mg/kg body weight) and high-dose tiliroside (300 mg/kg body weight) groups were lower than that of the hyperuricemic control group (Table 1).

**Figure 4:**
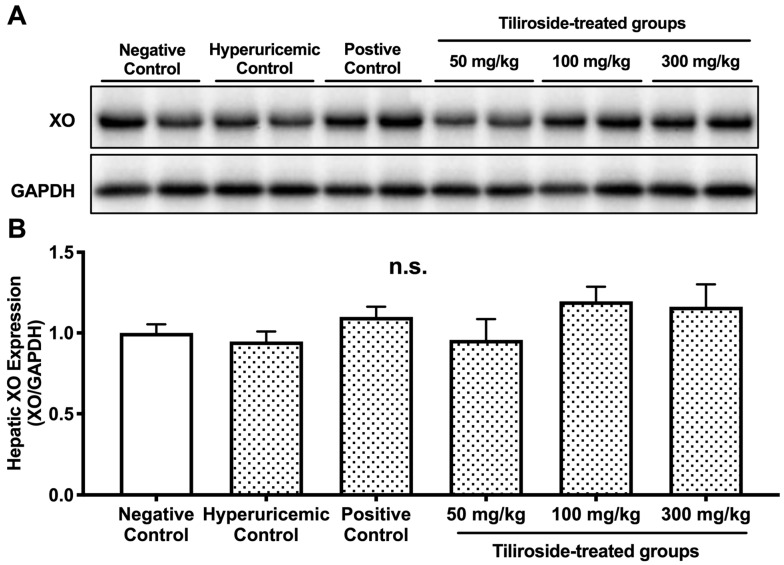
Western blot analysis of hepatic XO protein expression in hyperuricemic model mice treated with tiliroside. Panel (**A**): Representative blot images of XO and GAPDH proteins in liver samples from each group. Panel (**B**): Quantification of XO protein levels normalized to GAPDH and expressed as relative ratios. Allopurinol (10 mg/kg body weight) was used as the positive control. Data are shown as mean ± SEM for four mice per group.

No statistically significant differences were observed in hepatic XO protein expression among the negative control, hyperuricemic control, allopurinol-treated, and tiliroside-treated groups at all tested doses (50, 100, and 300 mg/kg) (Figure 4).

### 2.5. Effect of Tiliroside on Renal UA Transporter Gene Expression in Hyperuricemic Model Mice

**Figure 5:**
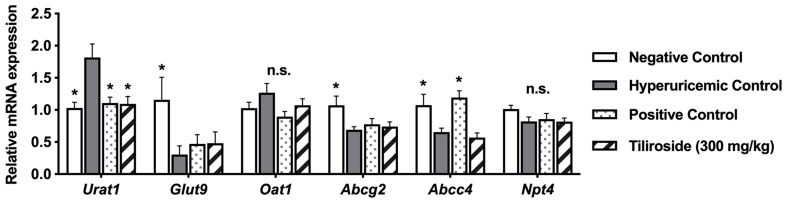
Effects of tiliroside on kidney mRNA expression of URAT1, GLUT9, OAT, ABCG2, ABCC4, and NPT4. Each value represents mean ± SEM for 8 mice. mRNA expression levels were normalized to GAPDH. Allopurinol (10 mg/kg body weight) was used as the positive control. For statistical significance, * *p* < 0.05 when the treated groups were compared with the hyperuricemic control group (Dunnett’s test).

The effects of tiliroside (300 mg/kg body weight) on mRNA levels of urate transporter 1 (URAT1), glucose transporter 9 (GLUT9), organic anion transporter 1 (OAT1), ATP-binding cassette sub-family G member 2 (ABCG2), ATP-binding cassette sub-family C member 4 (ABCC4), and Na^+^-dependent phosphate transporter 4 (NPT4) in hyperuricemic model mice are shown in Figure 5. The intraperitoneal injection of GMP and IMP significantly upregulated renal URAT1 expression in the hyperuricemic mice (Figure 5). Both allopurinol and tiliroside significantly downregulated renal URAT1 expression in the mice. GMP and IMP injection significantly downregulated renal GLUT9 and ABCG2 expression in the hyperuricemic mice. The same treatment downregulated expression of renal ABCC4 in the mice. Allopurinol significantly upregulated renal ABCC4 expression. There were no significant differences in the renal mRNA expression of OAT1 and NPT4 among the negative control, hyperuricemic control, allopurinol, and tiliroside administration groups.

## 3. Discussion

Tiliroside (kaempferol-3-O-(6′′-p-coumaroyl)-glucoside) is a glycoside of kaempferol and is found in rose hips, strawberries, and raspberries [8,9,10]. It has been reported that tiliroside is metabolized into its aglycone form, kaempferol, by intestinal microbiota [13]. In addition, astragalin (kaempferol-3-O-glucoside) and p-coumaric acid have also been identified as microbial metabolites of tiliroside (Figure 1A, showing the chemical structures of these compounds). In this study, we investigated the antihyperuricemic effects of tiliroside, kaempferol, astragalin, and p-coumaric acid through both in vitro and in vivo approaches.

Kaempferol exhibited strong XO inhibitory activity (IC_50_ = 3.9 µM, Figure 1B). In contrast, its glycosylated derivatives, tiliroside (kaempferol-3-O-(6′′-p-coumaroyl)-glucoside) and astragalin (kaempferol-3-O-glucoside) showed markedly lower activity, with IC_50_ values of >100 µM and 43 µM, respectively. These values are largely consistent with previous reports (tiliroside: IC_50_ >100 µM [23]; astragalin: IC_50_ = 49.46 µM [24]), supporting the reproducibility and reliability of the inhibitory effects in the present study. From these results, it was demonstrated that the direct inhibitory effect of tiliroside on XO is limited. Furthermore, these results suggest that glycosylation at the C-3 position of kaempferol suppress its XO inhibitory effect, possibly due to steric hindrance or reduced binding affinity to the active site of XO. Previous studies have also reported that the C-3 glycosides of quercetin, one of the major flavonoids, such as rutin (quercetin-3-O-rutinoside), quercitrin (quercetin-3-O-rhamnoside), and isoquercetin (quercetin-3-O-glucoside), as well as kaempferol-3-O-rutinoside, a C-3 glycoside of kaempferol, exhibit lower XO inhibitory activity compared to their respective aglycones [23,25]. These findings are consistent with the results of the present study. Tiliroside exhibited weaker activity than astragalin, indicating that the presence of the p-coumaroyl group further diminished its inhibitory effect. Additionally, p-coumaric acid alone showed weak activity (IC_50_ >100 µM), suggesting that this structural unit does not significantly contribute to XO inhibition. These findings demonstrate the importance of the hydroxyl group at the C-3 position of kaempferol in XO inhibition and suggest that structural modifications such as glycosylation and p-coumaroyl-type aromatic substitutions may reduce its inhibitory activity.

In this study, we assessed the potential of tiliroside, kaempferol, astragalin, and p-coumaric acid to suppress UA synthesis in AML12 hepatocytes. Allopurinol, a clinically utilized XO inhibitor, served as the reference compound. It markedly suppressed UA accumulation in a concentration-dependent manner, confirming the robustness of the assay system (Figure 2B). Tiliroside and kaempferol also led to a significant, dose-dependent decline in UA production. In comparison, the inhibitory effect of astragalin was less pronounced than that of tiliroside and kaempferol. In addition, p-coumaric acid exhibited no inhibitory effect at the concentrations up to 100 µM. Kaempferol exhibited a dose-dependent inhibitory effect, which was consistent with the results of the XO inhibition assay (Figure 1B). Although astragalin showed in vitro XO inhibitory activity, its inhibitory effect on UA production in the hepatocytes was significant only at 100 µM, indicating that its effect on the hepatocytes was weak. These results suggested XO inhibitory activity in vitro assay does not necessarily correspond to the suppression of UA production in AML12 cells. This inconsistency may be attributed to structural differences between kaempferol and astragalin. Astragalin is a glycoside of kaempferol, with a glucose moiety attached at the C-3 position, which likely affects its membrane permeability and intracellular bioavailability. Similar differences in inhibition of UA production in AML12 cells due to glycosylation have also been reported for quercetin and its glycosides (rutin, quercitrin, and isoquercetin), suggesting that glycosylation tends to reduce efficacy of UA suppression in the cells [26]. Nevertheless, tiliroside, a kaempferol-3-O-(6′′-p-coumaroyl)-glucoside, significantly and dose-dependently suppressed UA production in AML12 hepatocytes. Even when the UA precursor was changed from guanosine and inosine to xanthine, their effects on UA productivity in AML12 cells were significant and dose-dependent. Tiliroside decreased UA production (nmol/2 h/mg protein) from 278.4 ± 8.8 ^a^ at 0 µM to 82.7 ± 2.4 ^b^ at 10 µM, 45.4 ± 1.8 ^c^ at 30 µM, and 28.0 ± 1.2 ^d^ at 100 µM. Kaempferol also markedly suppressed UA production, with values of 236.0 ± 7.2 ^b^ at 10 µM, 101.1 ± 2.8 ^c^ at 30 µM, and 31.4 ± 0.5 ^c^ at 100 µM, compared to 271.1 ± 10.5 ^a^ at 0 µM. These reductions in UA production were statistically significant, as indicated by different letters (*p* < 0.05). These results suggest the following four possibilities: (1) tiliroside may have been partially deglycosylated during the 2-h incubation with hepatocytes in BSS; (2) tiliroside may have been deglycosylated after being taken up into the cells; (3) the presence of the p-coumaroyl group may have facilitated cellular uptake of tiliroside; and (4) tiliroside may have selectively inhibited xanthine dehydrogenase (XDH) rather than XO. Further studies are desirable to clarify these possibilities.

The doses of tiliroside used in the present study (50, 100, and 300 mg/kg) were selected based on our previous investigations employing similar hyperuricemic mouse models [27]. In those studies, polyphenolic compounds such as quercetin and isorhamnetin exhibited significant antihyperuricemic effects at doses ranging from 100 to 300 mg/kg. Therefore, we adopted a comparable dosing strategy to evaluate the efficacy of tiliroside. The highest dose (300 mg/kg) was chosen to ensure a robust pharmacological response, while the lowest dose (50 mg/kg) was included to determine whether tiliroside could exert beneficial effects even at a minimal concentration. In the purine-induced hyperuricemia model, oral administration of tiliroside (300 mg/kg) and the reference drug allopurinol (10 mg/kg) significantly attenuated the elevation of plasma UA levels (Figure 3). These results demonstrate, for the first time, the hypouricemic efficacy of tiliroside in vivo. Tiliroside at 300 mg/kg, like allopurinol, significantly reduced hepatic UA content and XO activity in the hyperuricemic model mice (Table 1). The combination of GMP and IMP did not significantly alter hepatic XO protein expression compared to the negative control group (Figure 4), and neither tiliroside nor allopurinol affected XO protein levels. These findings indicate that the antihyperuricemic action of tiliroside in GMP/IMP-induced hyperuricemic model mice is primarily attributable to its ability to inhibit hepatic XO enzymatic activity, rather than modulating XO protein expression. Considering that tiliroside did not directly inhibit XO in the enzyme inhibition assay, and that its aglycone, kaempferol, has been reported to reduce blood UA levels and hepatic XO activity in hyperuricemic model mice [28], it is possible that the decreased hepatic XO activity observed in the model mice in this study is attributable to kaempferol, which may have been derived through intracellular deglycosylation or metabolism by gut microbiota. Further studies are needed to determine whether the observed decrease in hepatic XO activity in this model is due to tiliroside or its metabolite, kaempferol. In contrast, tiliroside at the middle dose (100 mg/kg body weight) reduced hepatic UA levels without affecting plasma UA levels (Figure 3 and Figure 4A), suggesting that its effect may be limited to a local (hepatic) level rather than exerting a whole-body hypouricemic action. Moreover, since no inhibition of XO activity was observed at this dose (Table 1), the reduction in hepatic UA levels may be attributed not only to the inhibition of XO but also to inhibition of XDH. In support of this, it has also been reported that quercetin and rutin inhibit both XO and XDH in the liver of hyperuricemic model mice [29]. Further studies, including the assessment of XDH activity, are warranted to clarify the underlying mechanism.

The kidney plays a central role in UA excretion. In humans, UA filtered by the glomerulus undergoes complex processes of reabsorption and secretion in the proximal tubules, resulting in the urinary excretion of approximately 10% of the filtered UA. URAT1 and GLUT9 mediate UA reabsorption via the apical and basolateral membranes of proximal tubular cells, respectively, whereas ABCG2, ABCC4, and NPT4, which are localized to the apical membrane, and OAT1, which is localized to the basolateral membrane, are involved in UA secretion into the urine [30]. Especially, URAT1 is regarded as a key transporter for urate reabsorption [2]. In the present study, treatment with tiliroside did not affect the mRNA expression levels of GLUT9, OAT1, ABCG2, ABCC4, or NPT4 (Figure 5). However, tiliroside administration significantly decreased the mRNA expression level of URAT1. Previous reports have shown that the UA-lowering effects of baicalein, a type of flavone, and fisetin, a flavonol found in various fruits and vegetables, are partly mediated by promoting UA excretion through the downregulation of URAT1 expression in the kidney [16,17]. Therefore, this result suggests that tiliroside may exert a uricosuric effect by suppressing UA reabsorption through the downregulation of URAT1 expression in the kidney. Furthermore, administration of GMP and IMP significantly reduced the mRNA expression level of GLUT9 (Figure 5), which may represent a homeostatic response to elevated plasma UA levels induced by these purine nucleotides. Additionally, allopurinol decreased the mRNA expression level of ABCC4, suggesting that allopurinol may also exert a uricosuric effect through modulation of ABCC4 expression. Further studies are needed to elucidate the mechanisms by which tiliroside and allopurinol promote UA excretion in the kidney.

In the present study, tiliroside exhibited an antihyperuricemic effect in the hyperuricemic model mice. As above-mentioned, tiliroside is a natural flavonoid found in strawberries, raspberries, and rose hips, and has a long history of consumption as a food ingredient. Furthermore, considering that tiliroside has been reported to be non-toxic and non-mutagenic [31], and that toxicity issues have been reported for existing clinical drugs used to treat hyperuricemia [5,6,7], tiliroside is suggested to be a promising candidate for the prevention and improvement of hyperuricemia.

Future studies should focus on pharmacokinetic profiling, metabolite identification, and clinical validation to explore the feasibility of tiliroside as a functional food ingredient or nutraceutical for hyperuricemia management. In particular, evaluating its efficacy in human subjects and clarifying the contribution of microbial metabolism to its bioactivity will be essential.

In summary, tiliroside emerges as a safe and effective compound with multifaceted mechanisms of action, warranting further investigation toward its practical application in dietary strategies for hyperuricemia control.

## 4. Materials and Methods

### 4.1. XO Inhibition Assay In Vitro

XO inhibitory activity assay of tiliroside, kaempferol, astragalin, p-coumaric acid, and allopurinol in vitro was conducted according to our previously described procedure [26]. Tiliroside was obtained from ALB Materials Inc. (Henderson, NV, USA); astragalin from Extrasynthese (Lyon, France); kaempferol from Sigma-Aldrich (St. Louis, MO, USA); p-coumaric acid from Nacalai Tesque, Inc. (Kyoto, Japan); and allopurinol from FUJIFILM Wako Pure Chemical Corporation (Osaka, Japan). All compounds used in this study were commercially available synthetic standards and were not extracted from plant materials. Allopurinol is a synthetic XO inhibitor that is widely used in the clinical treatment of hyperuricemia and gout. In this study, it was employed as a positive control in both in vitro and in vivo experiments. Prior to the assay, the compounds were freshly dissolved in a mixture of 30% dimethyl sulfoxide (DMSO; FUJIFILM Wako) and 70% phosphate buffer (100 mM, pH 7.5) to prepare test solutions at final concentrations ranging from 0 to 100 µM. In each well of a 96-well microplate, 50 µL of the test solution was mixed with 60 µL of buffer and 30 µL of xanthine oxidase (7.8 mU/mL, bovine milk-derived; Sigma-Aldrich). After preincubation at 37 °C for 10 min, the reaction was initiated by adding 60 µL of xanthine substrate (150 µM; FUJIFILM Wako). Absorbance at 295 nm was monitored for 30 min at 37 °C using a Spark 10 M microplate reader (Tecan Group Ltd., Männedorf, Switzerland). XO inhibitory activity was expressed as percentage inhibition, calculated using the formula Inhibition (%) = 100 × [1 − (B/A)], where A and B represent the absorbance increase in the absence and presence of the test compound, respectively. All measurements were performed in triplicate.

### 4.2. Determination of UA Production by AML12 Cells

AML12 hepatocytes used in this study were acquired from ATCC (CRL-2254), Manassas, VA, USA. AML12 cells were maintained in DMEM/F-12 (Life Technologies, Grand Island, NY, USA) supplemented with 10% (*v*/*v*) fetal bovine serum (FBS; Hyclone, Logan, UT, USA), recombinant human insulin (5 µg/mL), human transferrin (5 µg/mL), and dexamethasone (40 ng/mL), each obtained from FUJIFILM Wako, as well as selenium (3 ng/mL, Sigma-Aldrich) and penicillin–streptomycin mixed solution (100 U/mL penicillin and 100 µg/mL streptomycin, Nacalai Tesque), under a humidified atmosphere of 5% CO_2_ at 37 °C. Culture of AML12 hepatocytes and measurement of UA production were conducted following a previously reported protocol [21,32].Cells were seeded at a density of 1.0 × 10^5^ cells per well in 24-well plates and cultured for 72 h in complete medium, followed by 24 h in serum-free DMEM/F-12. After serum deprivation, cells were rinsed once with calcium- and magnesium-free phosphate-buffered saline [PBS (–)] and incubated in 200 µL of balanced salt solution (BSS) containing 188 mM NaCl, 5 mM KCl, 1 mM MgCl_2_, 0.8 mM CaCl_2_, 25 mM NaHCO_3_, 1 mM NaH_2_PO_4_, 10 mM HEPES, and 5 mM glucose [32]. To stimulate UA production, BSS was supplemented with 100 µM each of guanosine and inosine (both from Sigma-Aldrich), referred to as GI mixture. Test compounds were added to the GI mixture and included allopurinol (0, 0.1, 0.3, or 1 µM), tiliroside, kaempferol, astragalin, and p-coumaric acid (each at 0, 10, 30, or 100 µM). The final concentration of DMSO in all samples was adjusted to 0.15%. After a 2-h incubation, 200 µL of the reaction mixture was collected for UA quantification. The UA concentration in BSS was used as an indicator of UA production [21]. Subsequently, cells were washed with PBS (–) and lysed in 300 µL of buffer containing 50 mM Tris and 1 mM sodium phosphate (pH 7.5). Lysates were sonicated and centrifuged (12,000× *g*, 5 min, 4 °C), and the supernatants were used for protein quantification using the Pierce™ BCA Protein Assay Kit (Thermo Fisher Scientific Inc., Waltham, MA, USA). UA levels were measured using the uricase-based Uric Acid C-Test Wako (FUJIFILM Wako). UA production was expressed as nanomoles per 2 h per milligram of cellular protein (nmol/2 h/mg protein).

### 4.3. Assessment of Cell Viability

The viability of AML12 cells was determined using the WST-8 assay provided in the Cell Counting Kit-8 (Dojindo Laboratories, Kumamoto, Japan). Cells were initially seeded at 5 × 10^3^ cells per well in 96-well plates and maintained in DMEM/F-12 medium containing 10% (*v*/*v*) FBS for 72 h. To induce serum deprivation, the medium was replaced with serum-free DMEM/F-12, and cells were incubated for an additional 24 h. After this pre-treatment phase, cells were gently rinsed with BSS and then exposed to GI mixture, either alone or in combination with tiliroside, kaempferol, astragalin, or p-coumaric acid at concentrations of 0, 10, 30, or 100 µM. The treatment lasted for 2 h. Subsequently, cells were washed again with BSS and incubated with the WST-8 reagent for 1 h. Absorbance was measured at 490 nm using a microplate reader (MTP-310Lab; CORONA ELECTRIC Co., Ltd., Hitachinaka, Japan). Cell viability was normalized to the DMSO control (0 µM), which was set at 100%.

### 4.4. Animal Experiments

Male ICR mice at 4 weeks of age were obtained from Charles River Japan, Inc. (Yokohama, Japan). were housed in plastic cages under controlled conditions with a 12-h light-dark cycle (dark phase of 18:00–6:00) and constant temperature (22 °C). The mice were housed in groups of four mice for 7 days to acclimatize to the environment and maintained on tap water and regular diet (CRF-1, Oriental Yeast Co., Tokyo, Japan) ad libitum. Animal procedures were approved by the Animal Research Committee of Utsunomiya University (approval number: A14-0017) and conducted in accordance with its institutional guidelines for animal experiments.

The antihyperuricemic effect of tiliroside was evaluated using mice with purine-induced hyperuricemia, following a previously described procedure [21]. After a one-week acclimation period, the animals were assigned to six experimental groups in a manner that ensured similar average body weights across groups: a negative control group (*n* = 8), a hyperuricemic model group (*n* = 10), an allopurinol-treated group (10 mg/kg, *n* = 8; positive control), and three tiliroside-treated groups receiving low (50 mg/kg, *n* = 8), middle (100 mg/kg, *n* = 8), or high (300 mg/kg, *n* = 8) doses. The average body weight of mice at the time of group assignment was as follows: negative control group, 28.4 ± 0.6 g; hyperuricemic model group, 28.4 ± 0.7 g; allopurinol-treated group, 28.5 ± 0.4 g; tiliroside low-dose group (50 mg/kg), 28.5 ± 0.4 g; middle-dose group (100 mg/kg), 28.6 ± 0.4 g; and high-dose group (300 mg/kg), 28.6 ± 0.4 g, indicating that all groups were assigned with comparable baseline body weights. Allopurinol and tiliroside were suspended in 0.5% sodium carboxymethylcellulose (CMC-Na, FUJIFILM Wako) and administered orally once daily for three consecutive days. The test compounds were suspended in 0.2 mL of 0.5% CMC-Na based on the average body weight of each group. The actual volume administered was adjusted according to the individual body weight of each mouse. Prior to each administration, the mice were fasted for four hours. The negative and hyperuricemic control groups received the vehicle (0.5% CMC-Na) alone. On the third day of treatment, hyperuricemia was induced by intraperitoneal injection of guanosine monophosphate (GMP) and inosine monophosphate (IMP, both from Tokyo Chemical Industry, Tokyo, Japan), each at a dose of 300 mg/kg body weight, dissolved in PBS (–). GMP and IMP were dissolved in 0.2 mL of PBS (–) based on the average body weight of all mice. The injection volume was similarly adjusted according to each mouse’s body weight. These injections were performed one hour after the final oral administration of the test compounds or vehicle. The negative control group received PBS (–) alone. One hour after GMP and IMP injection, blood samples were collected from the inferior vena cava under isoflurane anesthesia using heparinized microtubes. The liver and kidneys were then excised. Blood samples were centrifuged at 5000× *g* for 10 min at 4 °C to obtain plasma, which was stored at −80 °C until analysis.

### 4.5. Liver Preparation for Analysis

Liver samples preparation was performed according to the methods previously described [27]. Each portion of the liver tissue was processed for a specific analysis. One portion was homogenized in ice-cold 100 mM Tris-HCl buffer (pH 7.5), followed by sonication and centrifugation at 10,000× *g* for 5 min at 4 °C. The resulting supernatant was collected for the determination of UA levels. Another portion was treated similarly using Tris-HCl buffer containing 1 mM EDTA-2Na, and the supernatant was used to measure XO activity. The final portion was homogenized in ice-cold RIPA lysis buffer (Nacalai Tesque) and centrifuged under the same conditions, and the resulting supernatant was used for Western blot analysis.

### 4.6. Quantification of UA in Plasma and Liver Tissue

UA concentrations in plasma and liver homogenates were determined using the uricase-based Uric Acid C-Test Wako. Protein concentrations in the liver samples were measured with the Pierce™ BCA Protein Assay Kit. Liver UA levels were expressed as milligrams per gram of liver protein (mg/g protein).

### 4.7. Assessment of Liver XO Activity

Hepatic XO activity was assessed using a previously reported method [28], with slight modifications to accommodate 96-well microplate measurements [33]. In brief, 40 µL of liver homogenate was mixed with 30 µL of ice-cold 100 mM Tris-HCl buffer (pH 7.5) containing 1 mM EDTA-2Na in each well. The enzymatic reaction was initiated by adding 180 µL of xanthine solution (150 µM in the same buffer), and the absorbance at 295 nm was monitored at 37 °C for 30 min using a Spark 10 M microplate reader. UA formation was quantified based on a standard curve generated with known concentrations of UA. Protein content in the homogenates was determined using the Pierce™ BCA Protein Assay Kit, and XO activity was expressed as micromoles of UA produced per minute per milligram of protein (µmol UA/min/mg protein).

### 4.8. Western Blot Analysis

Western blotting was performed according to a previously reported protocol [34], with minor modifications. To ensure representative sampling, four mice per group were selected based on plasma UA levels approximating the group mean. Liver proteins were extracted and separated by SDS-PAGE, then transferred onto PVDF membranes. The membranes were blocked for 1 h at room temperature with 5% bovine serum albumin dissolved in Tris-buffered saline containing 0.1% Tween-20 (Sigma-Aldrich), referred to as TBST. Primary antibodies against XO and GAPDH (both from Santa Cruz Biotechnology, Inc., Dallas, TX, USA) were applied and incubated overnight at 4 °C. After thorough washing with TBST, the membranes were incubated with a secondary antibody: horseradish peroxidase-conjugated anti-mouse IgG antibody (GE Healthcare, Buckinghamshire, UK), and signals were visualized using the Amersham™ ECL™ detection system (GE Healthcare).

### 4.9. Real-Time Quantitative PCR Analysis

To assess renal gene expression, four mice per group were selected based on plasma UA levels approximating the group mean. Total RNA was extracted from kidney tissues of the negative control, hyperuricemic model, allopurinol-treated, and high-dose tiliroside-treated groups using the Trizol-chloroform method (Thermo Fisher Scientific). Reverse transcription was performed with 1 µg of RNA using iScript reverse transcriptase (Bio-Rad, Hercules, CA, USA). Quantitative PCR was conducted using the MyiQ2 real-time PCR system (Bio-Rad), with GAPDH as the reference gene. Primer sequences were adopted from previously validated sources [35,36,37] and are listed in Table 2.

### 4.10. Statistical Analysis

All experimental data are presented as mean ± SEM. Statistical comparisons for cell viability and UA production in AML12 cells were conducted using one-way ANOVA, followed by Tukey’s post hoc test. For animal studies, one-way ANOVA with Dunnett’s multiple comparisons test was applied to evaluate differences between treatment groups and the control. *p* values less than 0.05 were considered statistically significant. Data from the in vitro XO inhibition assay are shown as mean values from three independent experiments. IC_50_ values were calculated by nonlinear regression analysis using dose–response curves generated from at least six concentrations of each compound. All statistical analyses were performed using GraphPad Prism version 10 (GraphPad Software, San Diego, CA, USA).

### 4.11. Use of AI-Assisted Tools in Manuscript Preparation

In the process of manuscript preparation, an AI-based language model (Microsoft Copilot, powered by GPT-4 Turbo, Microsoft Corporation, Redmond, WA, USA) was used to assist in refining English grammar, improving sentence clarity, and rephrasing technical descriptions. All scientific content, data interpretation, and conclusions were developed independently by the authors.

## 5. Conclusions

In this study, we investigated the antihyperuricemic potential of tiliroside, a flavonoid glycoside naturally found in strawberries, raspberries, and rose hips. Tiliroside reduced UA biosynthesis in hepatocyte cultures and attenuated the elevation of plasma and hepatic UA levels in mice with purine-induced hyperuricemia. These effects may be partially attributed to the modulation of hepatic XO activity. Additionally, a decrease in renal URAT1 mRNA expression was observed in tiliroside-treated mice, which may suggest a possible involvement of renal UA transport mechanisms; however, further studies are required to confirm functional implications.

While tiliroside is naturally found in strawberries, raspberries, and rose hips, the compound used in this study was a commercially synthesized product, ensuring consistent purity and reproducibility across experiments.

Given its natural occurrence in commonly consumed edible plants and its favorable safety profile demonstrated in both in vitro and in vivo models [13], tiliroside appears to be a safe dietary compound. Although no adverse effects were observed at doses up to 100 mg/kg in mice, further studies are needed to evaluate the safety of higher doses and long-term administration.

Taken together, although additional research is necessary to clarify the underlying biological processes, our findings suggest that tiliroside may act as a safe and functional dietary compound for supporting the prevention and management of hyperuricemia.

## Figures and Tables

**Table 1:** Hepatic UA levels and XO activity in hyperuricemic model mice following tiliroside administration.

Group	Hepatic UA Level (mg/g Protein)	Hepatic XO Activity (µmol UA/min/mg Protein)
Negative control	0.74 ± 0.07 *	0.56 ± 0.06 *
Hyperuricemic control	1.73 ± 0.23	0.95 ± 0.11
Positive control	0.60 ± 0.03 *	0.34 ± 0.04 *
Tiliroside (50 mg/kg)	1.30 ± 0.16	0.70 ± 0.08
Tiliroside (100 mg/kg)	1.13 ± 0.16 *	0.82 ± 0.06
Tiliroside (300 mg/kg)	0.79 ± 0.11 *	0.67 ± 0.07 *

Data are shown as mean ± SEM for 8–10 mice, with duplicate measurements per mouse. Allopurinol (10 mg/kg body weight) was used as the positive control. * *p* < 0.05 versus the hyperuricemic control group (Dunnett’s test).

**Table 2:** Primer sequences used for qRT-PCR analysis.

Gene	Forward Primer (5′→3′)	Reverse Primer (5′→3′)
Urat1	GCTACCAGAATCGGCACGCT	CACCGGGAAGTCCACAATCC
Glut9	GAGATGCTCATTGTGGGACG	GTGCTACTTCGTCCTCGGT
Abcg2	TAAATGGAGCACCTCAACCT	GAGATGCCACGGATAAACTG
Abcc4	TAATGGAAGCAGACAAGGCCCAGA	AGAGGCCAGTGCAGATACATGGTT
Npt4	TCTGCACCATTGCCTTGTCA	CAAATACCCATCTAGACAACACATCTTT
Oat1	GCCTATGTGGGCACCTTGAT	CTTGTTTCCCGTTGATGCGG
Gapdh	TGAGGCCGGTGCTGAGTATGT	CAGTCTTCTGGGTGGCAGTGAT

## Data Availability

The data obtained in this study will be made available by the corresponding author upon reasonable request, in accordance with applicable ethical and institutional guidelines.

## Abbreviations

ABCC4	ATP-binding cassette sub-family C member 4
ABCG2	ATP-binding cassette sub-family G member 2
BSS	balanced salt solution
CMC-Na	carboxymethyl cellulose sodium
DMSO	dimethyl sulfoxide
FBS	fetal bovine serum
GAPDH	glyceraldehyde-3-phosphate dehydrogenase
GLUT9	glucose transporter 9
GMP	guanosine 5′-monophosphate
IMP	inosine 5′-monophosphat
NPT4	Na^+^-dependent phosphate transporter 4
OAT1	organic anion transporter 1
PBS	phosphate-buffered saline
UA	uric acid
URAT1	urate transporter 1
XDH	xanthine dehydrogenase
XO	xanthine oxidase
